# Case report: A rare case of delayed drug-induced hyponatremia in recurrent upper tract urothelial carcinoma following GC and Tislelizumab treatment

**DOI:** 10.3389/fonc.2024.1528237

**Published:** 2025-01-13

**Authors:** Zhi-Jie Wang, Ying-Fang Nie, Shi-Bing Liang, Jing Zhou, Shu-Lan Hao, Li-Kun Liu

**Affiliations:** ^1^ Postdoctoral Research Station, The First Affiliated Hospital of Henan University of Chinese Medicine, Zhengzhou, China; ^2^ The Oncology Department, Shanxi Hospital of Traditional Chinese Medicine, Taiyuan, China; ^3^ Clinical Study Center, Affiliated Hospital of Shandong University of Traditional Chinese Medicine, Jinan, China; ^4^ Centre for Evidence-Based Chinese Medicine, Shandong University of Traditional Chinese Medicine, Jinan, China; ^5^ Postdoctoral Research Station, Shandong University of Traditional Chinese Medicine, Jinan, China

**Keywords:** drug-induced hyponatremia, upper tract urothelial carcinoma, cisplatin, gemcitabine, Tislelizumab, borderline or mildly abnormal kidney function

## Abstract

Drug-induced hyponatremia is an adverse reaction with accelerated electrolyte disturbance. This study reported a rare case of delayed hyponatremia in a 68year-old female with recurrent upper tract urothelial carcinoma after Gemcitabine plus Cisplatin (GC) and Tislelizumab treatment. She had left ureter surgery, recurrence a year later with mildly abnormal kidney function (glomerular filtration rate (GFR) was 54.9 ml/min). After the first cycle of GC plus Tislelizumab, severe hyponatremia leading to life-threatening conditions occurred eight days later. Hypothesizing Cisplatin as the cause, its usage was modified in the second cycle (40mg/day for three days). No severe hyponatremia followed. CT showed partial remission. From the third cycle, due to grade IV bone marrow suppression, she had Tislelizumab alone. Now, she is on 21-day Tislelizumab maintenance with a stable tumor status. Low-dose continuous Cisplatin may suit patients with borderline or mildly abnormal renal function (GFR: 40-60mL/min) better than single full-dose use. Tislelizumab alone for maintenance may be an option for those intolerant of chemotherapy. But Na^+^ decrease may be related to Tirelizumab or Gemcitabine, needing more clinical observation and experiments.

## Introduction

Due to its favorable tolerability profile, Gemcitabine plus Cisplatin (GC) has become a standard first-line chemotherapy regimen for urothelial carcinoma with an overall survival of 15.2 to 15.8 months (1). However, the susceptibility of chemotherapy to drug resistance necessitates the exploration of novel therapeutic strategies to improve patient outcomes and enhance survival times in this relatively uncommon condition, especially for patients with poor general condition or distant metastases. Recently, the discovery of programmed death 1 (PD-1) and associated research have revealed that immune evasion is crucial in the development of tumors. PD-1 and its ligand 1 (PD-L1) are involved in tumor cell immune escape. Immunotherapy targeting PD-1 and PD-L1, based on immune checkpoint inhibitors, provides patients with treatment options and has been effectively used for various malignancies, including urothelial carcinoma (2, 3). Platinum-based chemotherapy boosts the blocking of PD-1 or PD-L1 and induces immune regulatory effects (4). In the tumor microenvironment, the significant over expression of PD-1/PD-L1 shields tumor cells from apoptosis (5). Moreover, urothelial carcinoma has a relatively high mutational load, meaning more tumor antigens for the immune system to recognize, further backing immunotherapy application in it. By the Chinese Anti-Cancer Association Clinical Oncology Collaborative professional committee (CSCO) (Version 2023), immunotherapy including Pembrolizumab and Atezolizumab, has been recommended as a monotherapy or combined with chemotherapy for the first-line treatment of locally advanced or metastatic urothelial carcinoma (6).

Tislelizumab is a PD-1 monoclonal antibody widely used in the clinical treatment of various malignancies and has demonstrated good clinical efficacy, safety and economical efficiency (7). Its combo with GC has shown excellent clinical efficacy and tolerability in patients with various cancers including urothelial carcinoma (8–10). A Phase 2 trial on PD-L1-positive urothelial carcinoma patients during or after platinum-containing chemotherapy (n=113, median follow-up 9.4 months) has a 24% confirmed objective response rate (10 complete responses and 15 partial responses), 68% sustained response among responders, and median progression-free survival and overall survival were 2.1 and 9.8 months respectively (7). Another study on its first-line use with platinum-containing chemotherapy in advanced urothelial carcinoma (n=31) showed a median progression-free survival of 36.0 weeks in the combo group versus 29.0 weeks in the chemotherapy-alone group (11).

## Case report

**Figure 1:**
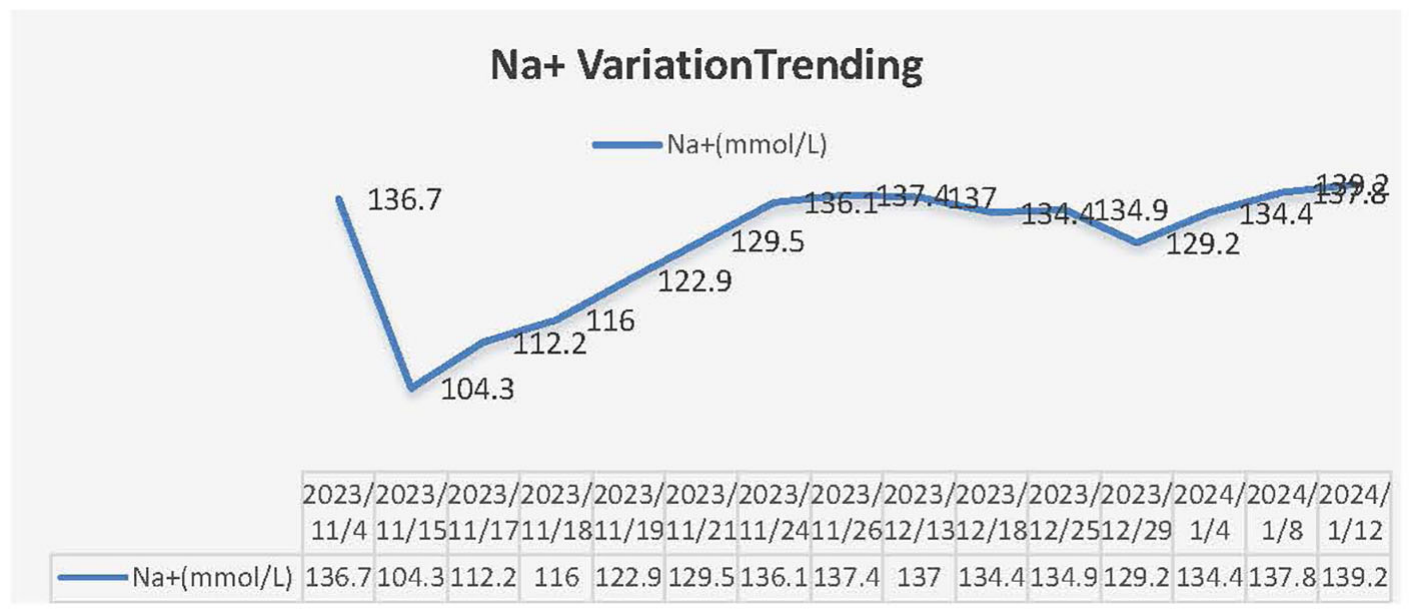
Na^+^ variation trending.

**Figure 2:**
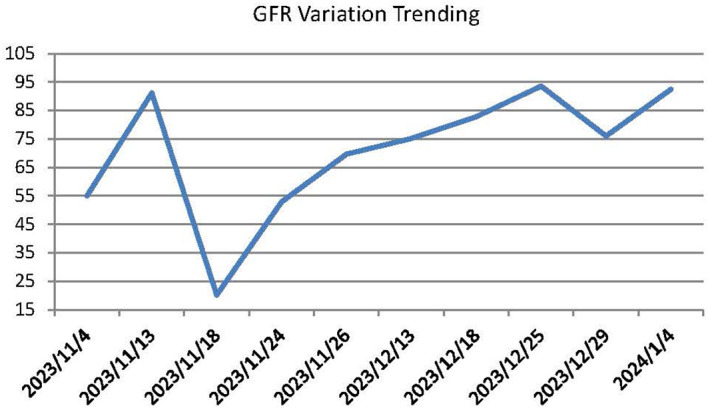
GFR variation trending.

This patient was a 68-year-old Chinese woman (168cm, 72.5kg) with a more than 1-year history of primary upper tract urothelial carcinoma. On 25^th^ October 2022, she underwent surgery on left ureter tumor plus left kidney and para-aortic lymph node dissection, which was staged as pT2N0M0, with a postoperative pathological diagnosis of high-grade invasive urothelial carcinoma and a size of 3×2cm. No definite nerve and vascular invasion was observed. No tumor cells were found in the left kidney, and no cancer was seen in the hilar vessels of the kidney, the stump of the ureter, the perirenal fat, or the para-aortic lymph nodes. She did not receive adjuvant chemotherapy following this surgery. A CT scan on 26^th^ October 2023 revealed thickening of the peritoneum and massive fluid accumulation in the abdomen and pelvis on. We have perfected the baseline assessment, and this patient’s general clinical condition is good with the score of Karnofsky Performance Status (KPS) of 90. The estimation of glomerular filtration rate (GFR) was 54.9 ml/min (normal range is from 90 to 125), and other items of kidney function were normal. And the urine volume was normal without other comorbidities, so we judged this patient could be able to tolerate immunotherapy and chemotherapy without other contraindications. On 7^th^ November 2023, she completed the first cycle of Tislelizumab plus GC, with cumulative dose of 200mg Tislelizumab, 800mg Gemcitabine, and 120mg Cisplatin. On 15^th^ November 2023, the patient complained of fatigue, poor appetite and dizzy. She gradually developed apathetic consciousness, slow response, and poor speech, with symptoms progressively worsening. The nuclear magnetic resonance of brain showed normal, and we obtained peripheral blood samples from this patient to test electrolyte, showing that Sodium went down from 136.7 mmol/L (4^th^, November) to 104.3 mmol/L (15^th^, November) (normal range is from 137 to 147 mmol/L). After intravenous infusion of concentrated saline, sodium levels began to normalize, and the patient’s overall condition gradually improved. (See **Figure 1** Na^+^ variation trending). During this process, there were relatively obvious changes in the GFR of this patient (See **Figure 2**). On 14^th^ December 2023, she started the second cycle of Tislelizumab plus GC with the same cumulative dose of 200mg Tislelizumab, 800mg Gemcitabine, and 120mg Cisplatin. Considering the severe hyponatremia that occurred after the first cycle treatment, we have modified the usage method of Cisplatin the second time as 40mg per day for three consecutive days. A CT re-examination on 4^th^ January 2024 indicated that the tumor had shrunk (1.9×1.8cm) and the ascites and pelvic effusion had completely disappeared, showing partial remission. No severe hyponatremia occurred after subsequent treatments. However, due to the occurrence of grade IV bone marrow suppression after the second cycle treatment, this patient discontinued chemotherapy starting from the third cycle and received Tislelizumab immunotherapy alone. Until now, she still administered for maintenance Tislelizumab treatment once every 21 days with a stable tumor status.

## Discussion

This case report describes a rare case of delayed electrolyte disturbance in a patient with recurrent upper tract urothelial carcinoma after GC and Tislelizumab treatment.

Urothelial carcinoma ranks sixth among common tumors in developed countries, among which, upper tract urothelial carcinoma accounts for 5-10%, and its annual incidence rate is about 2 cases per 100 000 inhabitants (1). Despite being a relatively rare disease, its global incidence and fatality rates continue to rise annually, according to estimates from the American Cancer Society in 2023, the incidence of cases in the kidneys/renal pelvis and ureters/other urinary organs was 81,800 and 4470, respectively, with mortality rates of 14,890 and 990, both showing an increase compared to previous years (12). UTUC is more vulnerable to infiltration and growth because of the disease’s hazy early symptoms and the thin muscular layer of the ureter and renal pelvis compared to that of the bladder. Approximately two-thirds of patients with UTUC already have muscle layer infiltration upon first diagnosis (1), and the postoperative survival rate of patients with muscle-invasive UTUC is reportedly poor (13, 14).

Electrolyte disturbances caused by Tislelizumab and Gemcitabine have not been reported. The incidence rate of hyponatremia caused by Cisplatin is reported 4%, and most cases are mild (15), while severe case is rare (16). Three main reasons might cause the hyponatremia by Cisplatin: first, dilutive hyponatremia caused by hydration; second, gastrointestinal dysfunction such as poor appetite, vomiting or diarrhea, leads to insufficient sodium intake or massive fluid loss; third, repeated application of cisplatin may cause kidney damage, which is manifested as the reabsorption of Na^+^ and Mg^2+^ plasma by the kidney. Obviously, this patient did not fit the ablove reasons. Renal salt wasting syndrome (RSWS) and syndrome of inappropriate antidiuretic hormone secretion (SIADH) are the rare reasons of severe hyponatremia associated with Cisplatin. RAWS is a condition in which the kidneys excrete an excessive amount of sodium into the urine. It leads to a negative sodium balance in the body. Some possible causes include certain kidney diseases, genetic disorders that affect the kidneys’ ability to reabsorb sodium, and some medications that can interfere with normal kidney function. Symptoms often include weakness, fatigue, hypotension, and in severe cases, it can lead to shock. Treatment usually focuses on replenishing the lost sodium and fluids. This may involve intravenous administration of saline solutions and careful monitoring of fluid and electrolyte balance. SIADH is a condition in which the body makes too much antidiuretic hormone (ADH). ADH is a hormone that helps the kidneys control the amount of water in the body. When there is too much ADH, the kidneys reabsorb too much water, leading to a dilution of the blood and a low sodium level in the blood (hyponatremia). Causes can include certain cancers (such as small-cell lung cancer), central nervous system disorders, some medications, and lung diseases. Symptoms may include nausea, vomiting, headache, confusion, seizures, and in severe cases, it can lead to coma. Treatment often involves fluid restriction and addressing the underlying cause. RAWS is different from the SIADH. In SIADH, there is water retention and dilutional hyponatremia, while in RAWS, sodium is lost inappropriately, and the body may also lose water as a result.

For patients with lowered GFR, the reduction of renal perfusion will damage the function of renal tubules. The reabsorption function of renal tubules for sodium depends on normal energy supply and appropriate blood flow environment. When these conditions are changed due to the decrease of GFR, the ability of renal tubules to reabsorb sodium will decline, thus leading to the loss of salt and triggering RAWS. On the other hand, if the occurrence of RAWS, the massive loss of salt and water could lead to a reduction in the effective circulating blood volume. It would cause a decrease in renal perfusion pressure, and subsequently, the GFR could be lowered. Because GFR is directly related to renal perfusion pressure, and renal perfusion pressure is an important component of the effective filtration pressure of the glomerulus. When the blood volume decreases, the blood pressure of the renal artery drops, and the blood pressure of the glomerular capillaries also decreases accordingly. According to the calculation formula of GFR (GFR=κf×(PGC - PBS - πGC) (κf is the filtration coefficient, PGC is the hydrostatic pressure of the glomerular capillaries, PBS is the pressure inside the renal capsule, and πGC is the plasma colloid osmotic pressure), the decrease in the hydrostatic pressure of the glomerular capillaries will result in a reduction in GFR, forming a vicious cycle. And also, when GFR decreases, the filtration of water and sodium by the kidneys is reduced (17). In SIADH, due to the inappropriate high-level secretion of antidiuretic hormone (ADH), it will cause an increase in the reabsorption of water by the renal tubules. The combined effect of these two situations will make the situation of water retention in the body even more serious. For example, the reduction of GFR reduces the filtration of water to some extent, and SIADH further causes the water that should be excreted to be overly reabsorbed by the renal tubules, thus aggravating symptoms such as hyponatremia and edema (18, 19). Research demonstrated that RAWS always occur within a few days to several months after Cisplatin exposure, and recovery can take place within a few days to several months, or it may persist. SIADH, on the other hand, has an earlier onset and can occur within the first two days after cisplatin administration. The blood sodium level returns to normal rapidly after the causative drug is discontinued (20, 21). Therefore, in light of the relevant characteristics of this patient, we mainly consider Cisplatin-induced RAWS.

There is also a particularly rare cause that may be related to renal salt wasting syndrome (RSWS). In RSWS, the kidney excretes too much sodium and some drugs have been reported lead to excessive sodium excretion. However, the decrease of Na^+^ cannot be ruled out to be related to Tirelizumab or Gemcitabine, and more clinical observation and experiments are needed.

## Conclusion

It is crucial to promptly identify symptoms and signs of hyponatremia during Cisplatin, Gemcitabine, and Tislelizumab anticancer treatment, especially in patients who have undergone urereral and nephrectomy with lowered GFR. Continuous use at a low dose may be more suitable for patients with borderline or mildly abnormal renal function (GFR: 40-60ml/min) than single use at a sufficient dose of Cisplatin. Maintenance immunotherapy with Tislelizumab alone may also be a good option for patients who cannot tolerate chemotherapy. Identifying the drug responsible for hyponatremia is essential to provide evidence for follow-up drug selection.

## Data Availability

The original contributions presented in the study are included in the article/**Supplementary Material**. Further inquiries can be directed to the corresponding author.
