# Supplementary material for: Case report: A rare case of delayed drug-induced hyponatremia in recurrent upper tract urothelial carcinoma following GC and Tislelizumab treatment
Source: Front Oncol. 2025 Jan 13;14:1528237. doi: 10.3389/fonc.2024.1528237 (PMC11769789; doi:10.3389/fonc.2024.1528237)
Supplement: Supplementary file 1 [file Table1.docx]

Supplementary tables

1. The amount of sodium ions on different dates

|  | Na+(mmol/L) |
| --- | --- |
| 2023/11/4 | 136.7 |
| 2023/11/15 | 104.3 |
| 2023/11/17 | 112.2 |
| 2023/11/18 | 116 |
| 2023/11/19 | 122.9 |
| 2023/11/21 | 129.5 |
| 2023/11/24 | 136.1 |
| 2023/11/26 | 137.4 |
| 2023/12/13 | 137 |
| 2023/12/18 | 134.4 |
| 2023/12/25 | 134.9 |
| 2023/12/29 | 129.2 |
| 2024/1/4 | 134.4 |
| 2024/1/8 | 137.8 |
| 2024/1/12 | 139.2 |

2. Change of the kidney function

| kidney function | UREA | Cr | URIC | CO2CP | GFR |
| --- | --- | --- | --- | --- | --- |
| 2023/11/4 | 4.91 | 92.6 | 339 | 27.16 | 54.9 |
| 2023/11/13 | 3.96 | 58.3 | 142 | 28.69 | 91.1 |
| 2023/11/18 | 8.17 | 212.8 | 231 | 19.24 | 20.1 |
| 2023/11/24 | 6.02 | 95.5 | 202 | 22 | 52.9 |
| 2023/11/26 | 3.59 | 76 | 193 | 24.98 | 69.6 |
| 2023/12/13 | 4.78 | 71.4 | 307 | 23.67 | 75.1 |
| 2023/12/18 | 5.9 | 65.9 | 204 | 26.07 | 82.8 |
| 2023/12/25 | 4.77 | 53.8 | 166 | 26.11 | 93.5 |
| 2023/12/29 | 7.6 | 70.7 | 213 | 24.87 | 76 |
| 2024/1/4 | 2.92 | 54.6 | 160 | 22.44 | 92.4 |
| unit | mmol/L | umol/L | umol/L | mmol/L | ml/min |
| reference range | 3.1-8.8 | 41-81 | 155-357 | 22-29 | 90-125 |
